# Employment Disruption and Financial Burden Associated with Gastrointestinal Cancers

**DOI:** 10.1245/s10434-025-17683-1

**Published:** 2025-06-26

**Authors:** Mujtaba Khalil, Selamawit Woldesenbet, Abdullah Altaf, Zayed Rashid, Shahzaib Zindani, Razeen Thammachack, Syed Husain, Timothy M. Pawlik

**Affiliations:** Department of Surgery, The Ohio State University Wexner Medical Center and James Comprehensive Cancer Center, Columbus, OH USA

**Keywords:** Cancer, Employment, Gastrointestinal, Financial burden

## Abstract

**Background:**

Understanding the extent of employment disruption and the financial strain faced by individuals with GI cancer is crucial to develop targeted support services. We sought to investigate employment disruption and financial burden among patients with gastrointestinal cancer (GI) diagnosis.

**Methods:**

Patients diagnosed with GI cancer were identified using the MarketScan database. Multivariable Cox proportional hazards models were used to evaluate the risk of employment disruption among patients with a GI cancer diagnosis.

**Results:**

A total of 11,832 individuals with GI cancer were included. Median patient age was 54 years (IQR: 49–59) with a majority of patients being male (*n*=7054; 59.6%). In the year following a GI cancer diagnosis, 13.8% (*n*=1638) of patients experienced employment disruption. Notably, individuals working in finance (HR 1.66, 95% CI 1.37–2.02), manufacturing (HR 1.19, 95% CI 1.01–1.42), and the transportation industry (HR 1.33, 95% CI 1.11–1.60) were at a higher risk of experiencing employment disruption. Patients with GI cancer had higher out-of-pocket (OOP) costs ($3250 [IQR: $1756–$5,255] vs. $453 [IQR: $142–$1189]), more workdays missed (50 [IQR: 28–$72] vs. 5 [IQR: 2–10]) (both *p *< 0.001) and a threefold higher risk of employment disruption (HR 3.16, 95% CI 2.96–3.38) compared with individuals without a GI cancer diagnosis.

**Conclusion:**

Patients with GI cancer are three times more likely to experience employment disruption and face significant financial burdens. Targeted measures, such as flexible work arrangements, job protection policies, and access to vocational rehabilitation services, are needed for patients with cancer.

**Supplementary Information:**

The online version contains supplementary material available at 10.1245/s10434-025-17683-1.

Over the last several decades, major advancements have been made in the treatment of patients with gastrointestinal (GI) cancer resulting in improved long-term outcomes.^1–3^ Unfortunately, accessing cancer treatment can be challenging for some patients due to substantial personal, social, and financial burdens.^4,5^ In addition, cancer therapy such as surgery, chemotherapy, and radiation therapy can often lead to considerable systemic toxicities.^6^ As a result, patients frequently experience physical challenges like pain, fatigue, and digestive issues, which severely impact their quality of life.^7^ Additionally, the emotional strain of battling cancer can lead to mental health issues such as depression and anxiety.^4^ The financial burden, including high medical costs and potential loss of income due to an inability to work, further exacerbates these difficulties for both patients and their families.^5^

Given that cancer treatment often involves complex and intensive medical interventions,^6^ many patients reduce their work hours or shift their career paths.^8^ For example, Nitecki et al. reported that gynecological cancers were linked to more than a threefold increase in the risk of employment disruption.^9^ Even years after treatment, patients wishing to return to work may face significant challenges in finding new employment.^9^ One U.S. population-based study estimated that annual productivity losses among working-age patients with cancer ranged from $9.6 to $16 billion.^10^ Unemployment can intensify the financial strain experienced by many patients diagnosed with cancer and may result in the loss of employer-sponsored health insurance, particularly for individuals with high healthcare costs.^8^ Beyond the financial impact, maintaining the ability to work after a cancer diagnosis may be crucial for emotional and social recovery, as well as enhance overall quality of life.^8,11^

Resuming employment after cancer treatment is associated with improved social functioning, greater financial security, better health, and higher self-esteem.^8^ Understanding the risk factors for employment disruption can help inform strategies to facilitate a return to work.^11^ However, employment disruption and financial hardships related to GI cancers remain ill-defined. Therefore, the current study sought to characterize employment patterns and financial challenges in the year following a GI cancer diagnosis.

## METHODS

### Data Source, Study Population, and Cohort Selection

The IBM MarketScan Commercial Claims and Encounters database was queried using International Classification of Diseases (ICD) codes to identify patients with a GI cancer diagnosis (Supplementary Table 1). This database includes commercial insurance claims from over 150 major employers and 300 employer-sponsored health plans, providing a national convenience sample of approximately 50 million patients under the age of 65 who have employer-sponsored health insurance.^12^ The analytic cohort comprised patients aged 18 and older who were newly diagnosed with esophageal, gastric, hepatic, biliary, pancreatic, colon, or rectal cancer between 2013 and 2020. The study included individuals with at least two outpatient or inpatient claims for GI cancer. The diagnosis date refers to the first claim of cancer in the medical records. Individuals who were employed full-time or part-time before their GI cancer diagnosis were included and followed for one year. Therefore, the cohort was restricted to individuals who maintained continuous insurance enrollment for 12 months before and after their cancer diagnosis. Individuals who did not receive any oncologic treatment (e.g. surgery, systemic, or local) during the year following the GI cancer diagnosis, as well as individuals who were not the primary beneficiaries of their health insurance plans, and patients with unknown employment information were excluded from the analytic cohort.

**Fig. 1:**
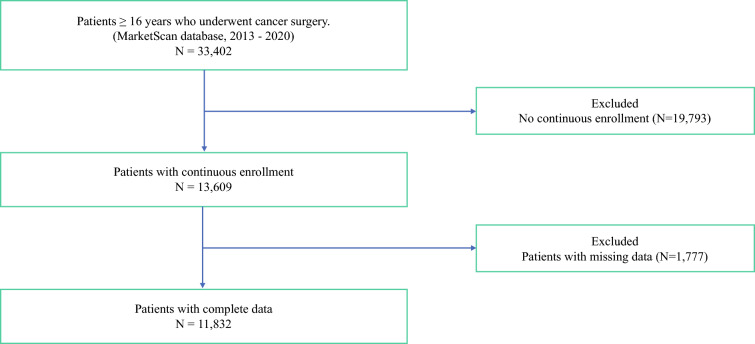
Flowchart depicting study cohort creation.

The control group consisted of individuals aged 18 years or older who had a billable encounter for a nonmalignant GI condition during the study period. The date of the billable encounter was defined as the index date. Entropy balancing was used to match individuals with GI cancer to available controls, ensuring that both cases and controls had index dates within three months of each other and comparable baseline characteristics. Baseline characteristics for which cases and controls were balanced included: age, insurance plan type, geographical region, year of diagnosis, CCI, index year, cancer type, and treatment. The models were adjusted for these variables. The control group was subjected to the same selection criteria as the GI cancer cohort, specifically 12 months of continuous coverage both before and after the index date, as well as full- or part-time employment. Additionally, individuals with missing data and those who were not the primary beneficiaries of their health insurance plans were excluded (Fig. 1). The current study followed Strengthening the Reporting of Observational Studies in Epidemiology (STROBE) reporting guidelines.^13^ The Institutional Review Board at Ohio State University approved this study and waived the requirement for informed consent since the data was deidentified.

### Outcomes of Interest

The primary outcome of interest was employment disruption in the year following GI cancer diagnosis. The IBM MarketScan database provided information on beneficiary employment status: active full-time, active part-time or seasonal, early retiree, Medicare-eligible retiree, retiree (status unknown), Comprehensive Omnibus Budget Reconciliation Act (COBRA) coverage (which allows eligible employees and their dependents to continue health insurance coverage after a job loss or a reduction in work hours), long-term disability, or other/unknown. For the 12 months following the GI cancer diagnosis and the index date for controls, employment status was recorded monthly. Employment was considered disrupted if there was any change from full-time to any other category or from part-time to any category other than full-time, as previously defined.^9,14^ Patients were censored after their first employment change.^9,14^

The secondary outcomes of interest included workdays missed and the financial burden among patients with GI cancer diagnosis. Workdays missed were calculated based on data from inpatient, outpatient, and emergency department visits.^12,15^ For inpatient visits, the total length of hospital stay was used to calculate missed workdays.^12,15^ Financial burden was assessed based on out-of-pocket costs (OOP) for hospitalizations, outpatient services/visits, and pharmacy prescriptions.^16,17^ Healthcare costs were adjusted for geographic variation using the wage index and for inflation using the Health Care Price Index.

### Covariates

Baseline covariates included patient age, sex, Charlson Comorbidity Index (CCI), insurance plan type, geographical region (i.e., Northeast, Northcentral, South, West), index year, and type of cancer. Patient health insurance types included comprehensive health coverage, exclusive provider organization (EPO), health maintenance organization (HMO), point-of-service (POS), preferred provider organization (PPO), and other (consumer-directed health plans [CDHP] and high-deductible health plans [HDHP]). Moreover, ICD-9/10 codes and Common Procedural Terminology codes were utilized to capture data on GI cancer treatment. To standardize treatment combinations across various GI cancers, treatments were classified as: (1) surgery only; (2) surgery combined with adjuvant therapy (any systemic or local treatment); and (3) chemotherapy, radiation, or chemoradiation alone (systemic or local therapy without surgery).^9^

### Statistical Analysis

Descriptive statistics were presented as median values with interquartile range (IQR) for continuous variables and as frequency and percentage (%) for categorical variables. Differences in baseline characteristics were assessed using the Kruskal–Wallis test for continuous variables and either the chi-square test or Fisher’s exact test for categorical variables. Cox proportional hazard models were used to identify factors associated with employment disruption among individuals with a GI cancer diagnosis; hazard ratios (HR) and 95% confidence intervals (CI) were reported. A multivariable generalized linear model with a gamma distribution and log link was employed to examine the healthcare burden. The models were adjusted for age, insurance plan type, geographical region, year of diagnosis, CCI, index year, cancer type, and treatment. Statistical tests were conducted using a two-tailed approach with a significance level of p<0.05. Analyses were performed using SAS 9.4 (SAS Institute).

## RESULTS

### Baseline Characteristics

Among the 52,012 individuals included in the analytic cohort, 22.7% (n=11,832) had a diagnosis of GI cancer (esophagus: *n*=680, 5.8%; stomach: *n*=703, 5.9%; liver: *n*=832, 7.0%; bile duct: *n*=224, 1.9%; pancreas: *n*=1096, 9.3%; colon: *n*=5464, 46.2%; rectum: *n*=2833, 23.9%) (Table 1). Median patient age was 54 years (IQR: 49–59) and most patients were male (*n*=28,955, 55.7%). Moreover, majority of the patients had a CCI score of ≤ 2 (*n*=50,695, 97.5%). Most of the patients resided in the South (*n*=24,276, 46.8%) or the North Central regions (*n*=12,205, 23.5%) followed by the Northeast (*n*=8094, 15.6%) and West regions (*n*=7354, 14.2%) of the United States. Moreover, 97.9% (n=50,902) of patients were employed full-time, while 2.1% (*n*=1110) had part-time employment. The most common type of health insurance was PPO (*n*=24,719, 47.8%), followed by HMO (*n*=6465, 12.5%), and POS (*n*=3797, 7.3%).

**Table 1:** Baseline characteristics of patients with and without a diagnosis of gastrointestinal cancer

Patient Characteristics	Total (*n*=52,012)	Gastrointestinal cancer diagnosis		*p* value
		No (*n*=40,180, 77.3%)	Yes (*n*=11,832, 22.7%)	
Age	54 (49–59)	54 (49–59)	54 (49–59)	0.722
Sex				<0.001
Male	28,955 (55.7)	21,901 (54.5)	7054 (59.6)	
Female	23,057 (44.3)	18,279 (45.5)	4778 (40.4)	
CCI				<0.001
≤2	50,695 (97.5)	40,121 (99.9)	10,574 (89.4)	
>2	1317 (2.5)	59 (0.1)	1,258 (10.6)	
Insurance plan				<0.001
Comprehensive	1298 (2.5)	1037 (2.6)	261 (2.2)	
EPO	403 (0.8)	276 (0.7)	127 (1.1)	
HMO	6465 (12.5)	5175 (13.0)	1290 (10.9)	
POS	3797 (7.3)	2973 (7.5)	824 (7.0)	
PPO	24,719 (47.8)	18,360 (46.0)	6359 (53.7)	
Others	15,078 (29.0)	12,107 (30.2)	2971 (25.1)	
Employment status				0.262
Active full time	50,902 (97.9)	39,338 (97.9)	11,564 (97.7)	
Part time	1,110 (2.1)	842 (2.1)	268 (2.3)	
Geographical region				<0.001
Northeast	8094 (15.6)	5980 (14.9)	2114 (17.9)	
North Central	12,205 (23.5)	9660 (24.0)	2545 (21.5)	
South	24,276 (46.8)	18,965 (47.2)	5311 (44.9)	
West	7354 (14.2)	5523 (13.7)	1831 (15.5)	
Metropolitan area				0.02
Nonmetropolitan	45,264 (87.0)	35,042 (87.2)	10,222 (86.4)	
Metropolitan	6748 (13.0)	5138 (12.8)	1610 (13.6)	
Year of diagnosis				0.098
2013	8776 (16.9)	6884 (17.1)	1892 (16.0)	
2014	7430 (14.3)	5771 (14.4)	1659 (14.0)	
2015	7681 (14.8)	5920 (14.7)	1761 (14.9)	
2016	6837 (13.2)	5273 (13.1)	1564 (13.2)	
2017	6191 (11.9)	4764 (11.9)	1427 (12.1)	
2018	4483 (8.6)	3423 (8.5)	1060 (9.0)	
2019	5720 (11.0)	4383 (10.9)	1337 (11.3)	
2020	4894 (9.4)	3762 (9.4)	1132 (9.6)	
1-year expenditures, $	3554	1994 (665–5,632)	1,17,916	<0.001
	(948–25,577)		(57,526–202,619)	
1 year OOP costs, $	719	453 (142–1,189)	3250	<0.001
	(210–2136)		(1,756–5255)	
Workdays missed	7 (3–21)	5 (2–10)	50 (28–72)	<0.001
Employment disruption	3656 (7.0)	2018 (5.0)	1638 (13.8)	<0.001

### GI Cancer Diagnosis and Employment Disruption

During the one-year follow-up period, 7.0% (n=3656) of individuals experienced employment disruption. Of note, patients with GI cancer were more likely to experience employment disruption (GI cancer: 13.8% vs. no GI cancer: 5.0%; *p *< 0.001) (Table 2).

**Table 2:** Baseline characteristics of individuals with gastrointestinal cancer, stratified by employment status

Patient Characteristics	Total (*n*=11,832)	Employment disruption		*p* value
		No (*n*=10,194, 86.2%)	Yes (*n*=1638, 13.2%)	
Age	54 (49–59)	53 (48–58)	56 (51–60)	< 0.001
Sex				0.399
Male	7054 (59.6)	6093 (59.8)	961 (58.7)	
Female	4778 (40.4)	4101 (40.2)	677 (41.3)	
CCI				< 0.001
≤2	10,574 (89.4)	9177 (90.0)	1397 (85.3)	
>2	1258 (10.6)	1017 (10.0)	241 (14.7)	
Insurance plan				<0.001
Comprehensive	261 (2.2)	212 (2.1)	49 (3.0)	
EPO	127 (1.1)	92 (0.9)	35 (2.1)	
HMO	1290 (10.9)	1103 (10.8)	187 (11.4)	
POS	824 (7.0)	745 (7.3)	79 (4.8)	
PPO	6359 (53.7)	4471 (53.7)	888 (54.2)	
Others	2971 (25.1)	2571 (25.2)	400 (24.5)	
Compensation				< 0.001
Salary	3623 (30.6)	3173 (31.1)	450 (27.5)	
Hourly wages	3781 (32.0)	3036 (29.8)	745 (45.5)	
Other	4,428 (37.4)	3,985 (39.1)	443 (27.0)	
Employment industry				< 0.001
Manufacturing	2350 (19.9)	1995 (19.6)	355 (21.7)	
Transportation	1596 (13.5)	1325 (13.0)	271 (16.5)	
Finance, real estate, insurance	1215 (10.3)	1020 (10.0)	195 (11.9)	
Services	2082 (17.6)	1830 (18.0)	252 (15.4)	
Other	1462 (12.4)	1247 (12.2)	215 (13.1)	
Unknown	3127 (26.4)	2777 (27.2)	350 (21.4)	
Geographical region				0.023
Northeast	2114 (17.9)	1817 (17.9)	297 (18.1)	
North Central	2545 (21.6)	2150 (21.2)	395 (24.1)	
South	5311 (45.0)	4594 (45.2)	717 (43.8)	
West	1831 (15.5)	1603 (15.8)	228 (13.9)	
Cancer type				< 0.001
Esophagus	680 (5.8)	549 (5.4)	131 (8.0)	
Stomach	703 (5.9)	584 (5.7)	119 (7.3)	
Liver	832 (7.0)	684 (6.7)	148 (9.0)	
Bile duct	224 (1.9)	182 (1.8)	42 (2.6)	
Pancreas	1096 (9.3)	847 (8.3)	249 (15.2)	
Colon	5464 (46.2)	4866 (47.7)	598 (36.5)	
Rectum	2833 (23.9)	2482 (24.4)	351 (21.4)	
Treatment				< 0.001
Systemic or local therapy only	4516 (38.2)	3749 (36.8)	767 (46.8)	
Surgery only	1881 (15.9)	1748 (17.2)	133 (8.1)	
Surgery with adjuvant	5435 (45.9)	4697 (46.0)	738 (45.1)	

Moreover, older patients (median age: 56 years [IQR: 51–60] vs. 53 years [IQR: 48–58]) and individuals with a high comorbidity burden (CCI >2: 14.7% vs. 10.0%) were more likely to experience employment disruption (both *p *< 0.001). In terms of compensation type and work industry, individuals working for hourly wages (disruption: 45.5% vs. no disruption: 29.8%) and in manufacturing (disruption: 21.7% vs. no disruption: 19.6%), transport (disruption: 16.5% vs. no disruption: 13.0%), and finance industries (disruption: 11.9% vs. no disruption: 10.0%) were more likely to experience employment disruption (all *p *< 0.001). Notably, patients who received only systemic or local therapy (disruption: 46.8% vs. no disruption: 36.8%) were also more likely to experience employment disruption, whereas individuals who underwent surgery only (disruption: 8.1% vs. no disruption: 17.2%) or surgery with adjuvant chemotherapy (disruption: 45.1% vs. no disruption: 46%) were less likely to experience employment disruption (all *p *< 0.001).

**Fig. 2:**
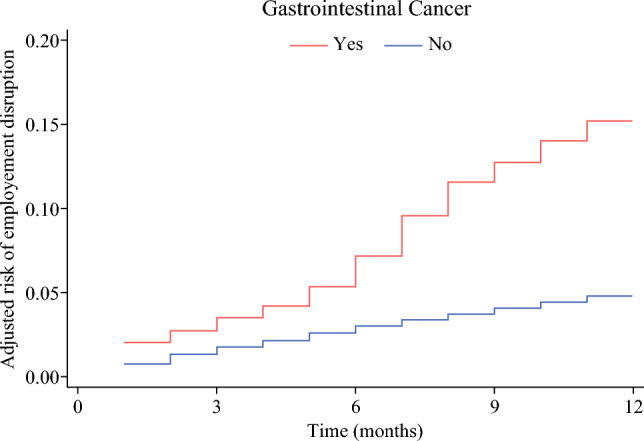
Adjusted risk of employment disruption in the first year following gastrointestinal cancer diagnosis.

Overall, GI cancer diagnosis was associated with a threefold risk (HR 3.16, 95% CI 2.96–3.38) of employment disruption (Fig. 2). Specifically, females (HR 1.12, 95% CI 1.01–1.24), and patients with high CCI score (HR 1.34, 95% CI 1.17–1.55) were at a higher risk of employment disruption. Additionally, compared with individuals employed in the services industry, patients working in finance (HR 1.66, 95% CI 1.37–2.02), manufacturing (HR 1.19, 95% CI 1.01–1.42), and transportation industries (HR 1.33, 95% CI 1.11–1.60) experienced higher risk of experiencing employment disruption. In terms of cancer site, patients with esophageal and gastric cancer (HR 4.08, 95% CI 3.37–4.94) had the highest risk of employment disruption, followed by those with HPB (HR 4.03, 95% CI 3.50–4.64) cancer and CRC (HR 2.78, 95% CI 2.55–3.04). Moreover, individuals who underwent surgery with adjuvant therapy (HR 1.92, 95% CI 1.59–2.32) and individuals who received any kind of systemic or local treatment without undergoing surgery (HR 2.13, 95% CI 1.75–2.59) were more likely to experience employment disruption than patients who underwent surgery alone (Table 3).

**Table 3:** Adjusted hazard ratios for employment disruption.

Characteristics	HR	95%, CI	*p* value
Age	1.04	1.03–1.04	< 0.001
Sex			0.032
Male	Ref	1.01–1.24	
Female	1.12		
CCI			
≤2	Ref		< 0.001
>2	1.34	1.17–1.55	
Compensation			
Salary	Ref		< 0.001
Hourly wages	1.65	1.47–1.87	
Employment industry			
Services	Ref		
Finance, real estate, insurance	1.66	1.37–2.02	< 0.001
Manufacture	1.19	1.01–1.42	0.047
Transportation	1.33	1.11–1.60	0.002
Other	1.25	1.04–1.52	0.017
Cancer site			
Esophagus and gastic	4.08	3.37–4.94	< 0.001
HPB	4.03	3.50–4.64	< 0.001
CRC	2.78	2.55–3.04	< 0.001
Treatment			
Surgery only	Ref		
Systemic or local therapy only	2.13	1.75–2.59	< 0.001
Surgery with adjuvant	1.92	1.59–2.32	< 0.001

### GI cancer Diagnosis Financial Burden and Workdays Missed

**Fig. 3:**
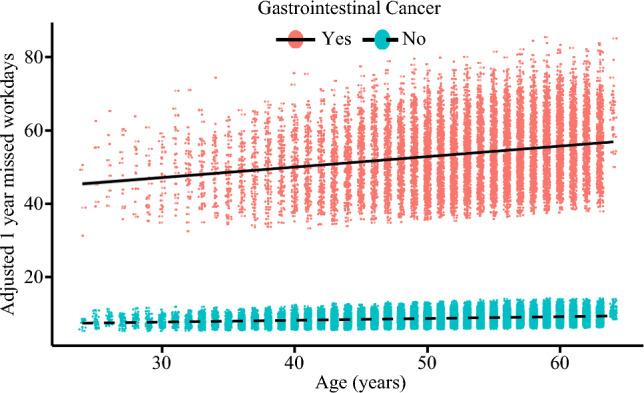
Scatterplot demonstrating the adjusted 1-year missed workdays, stratified by gastrointestinal cancer.

**Fig. 4:**
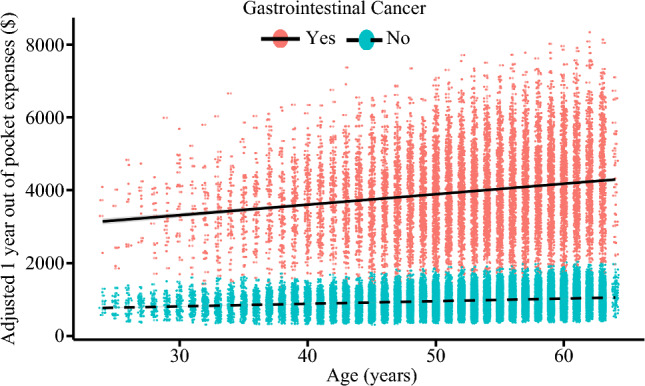
Scatterplot demonstrating the adjusted 1 year out of pocket costs, stratified by gastrointestinal cancer.

Overall, median total healthcare expenditures over one year were $3,554 (IQR: $948–$25,577). Median out-of-pocket expenses were $719 (IQR: $210–$2136), and median number of workdays missed was 7 (IQR: 3–21). Individuals with GI cancer had higher OOP costs (median: $3250 [IQR: $1756–$5255] vs. $453 [IQR: $142–$1189]) and more workdays missed (median: 50 [IQR: 28–$72] vs. 5 [IQR: 2–10]) (both *p *< 0.001) (Fig. 3 and Table 1). On multivariable analysis, GI cancer diagnosis was associated with increased financial burden (mean difference: $298, 95% CI $289–$307) and more missed workdays (mean difference: 80 days, 95% CI 70–100) (Figs. 3 and 4).

## DISCUSSION

Recent advancements in cancer care have lead to improved long-term outcomes.^3,18^ However, patients continue to encounter considerable personal, social, and financial challenges during their treatment journey.^5,19^ Of note, a cancer diagnosis increases the risk of employment disruption and the annual productivity losses among working-age individuals with cancer are estimated to be $16 billion.^20^ In turn, individuals with cancer who face employment disruption experience decreased social functioning, reduced financial security, and a lower overall quality of life.^8^ Nonetheless, employment disruption and financial burden among individuals with GI cancer remain ill-defined. Therefore, the current study was important as we specifically investigated employment disruption trends and financial burden faced by patients in the year following a GI cancer diagnosis. Of note, the risk of employment disruption increased threefold during the first year following a diagnosis of GI cancer. Additionally, patients with GI cancer experienced more missed workdays and faced greater financial burdens. These results highlight the need for targeted interventions, such as flexible work arrangements, job protection policies, and access to vocational rehabilitation services to better support patients with GI cancer.^21,22^

GI cancer has one of the highest incidence rates in the US, with 280,000 individuals diagnosed each year.^23^ A number of studies have examined personal and social challenges faced by individuals with GI cancer, such as emotional distress, difficulties with treatment adherence, and access to care.^11,24^ To the best of our knowledge, this is the first study to examine professional challenges, including disruption in employment status, missed workdays, and financial burdens. The current study noted an employment disruption rate of 13.8%, which is comparable with previous studies reporting on other acute and chronic diseases.^14,25^ For instance, Nitecki et al. reported that 21% of females with gynecologic cancers experienced a decrease in employment within the year following diagnosis.^14^ Similarly, Meernik et al. noted that 27.3% of breast cancer survivors either stopped working altogether or reduced their work hours due to cancer.^25^ The employment disruption reported in these previous studies was higher than in the current study, perhaps because these studies exclusively focused on female patients.^14,25^ Specifically, a recent meta-analysis reported that females are more likely to decrease work hours or completely stop working after a cancer diagnosis.^8^ The current study also highlighted that, following a GI cancer diagnosis, the risk of employment disruption increased threefold. Moreover, individuals earning hourly wages and those working in finance, manufacturing, and transportation industries are at higher risk of employment disruption after cancer treatment because these jobs often offer less flexibility, fewer benefits, and limited access to paid leave.^20^ Hourly positions and certain industries may also involve more physically demanding tasks or stricter attendance requirements, making it harder for cancer survivors to manage treatment side effects and maintain consistent employment.^20^ As a result, these workers are more vulnerable to losing their jobs or having to leave the workforce during or after their cancer treatment. Interestingly, individuals who received chemotherapy, radiation, or chemoradiation were also at a higher risk of employment disruption. Individuals undergoing chemotherapy or radiotherapy may have more advanced disease and be at a higher risk of fatigue and systemic symptoms, which may impair their ability to work.^6,8^ Of note, individuals who stop working within one year of their diagnosis often do so because of treatment-related symptoms.^25^ To this point, chemotherapy and radiotherapy are known to affect long-term physical and emotional well-being.^25^ In summary, understanding the risk factors for unemployment can help guide optimal treatment strategies to facilitate a return to work.^21^

Cancer treatment costs in the U.S. have been rising steadily, creating a significant financial burden for patients and straining the healthcare system.^26^ The U.S. healthcare system’s mix of private and public insurance has contributed to increased OOP expenses for cancer patients.^27^ In 2019, the national economic burden associated with cancer care was $21.09 billion, with $16.22 billion attributed to OOP costs.^27^ The current study highlights that cancer care often involves high OOP expenses, which combined with employment disruption and missed workdays, intensifies the financial burden. GI cancer treatments are particularly invasive and can result in severe side effects from chemotherapy and radiation therapy, requiring extensive recovery time.^19^ Moreover, the demanding nature of cancer treatment frequently leads to missed workdays due to hospital visits, readmissions, and overall reduced energy levels.^28^ The current dataset indicated that GI cancer patients miss more workdays and face higher OOP expenses, exacerbating their financial burden, especially with decreased income from employment disruption.

Reducing employment disruption and financial burden for cancer patients requires a multifaceted approach involving healthcare providers, employers, and policymakers.^29^ Healthcare teams should discuss potential employment challenges with patients early in the treatment process, helping individuals plan for workplace accommodations and leave options.^8^ For example, physicians should be aware of existing US government policies, such as the Family and Medical Leave Act and the Americans with Disabilities Act.^29^ A survey of patients with gynecologic cancer demonstrated that, although 74% of patients wanted to return to work, only 26% requested work accommodations and only 28% were aware of employer-specific policies regarding returning to work.^30^ Employers can also play a crucial role by offering flexible work arrangements, paid sick leave, and modified job duties to allow patients to continue working during treatment.^8^ Implementing comprehensive return-to-work programs that include physical, psychosocial, and educational components can facilitate smoother reintegration into the workplace.^8^ At a policy level, expanding leave policies beyond current limits and improving access to temporary disability insurance could better protect patients against lost wages and job loss.^25^ Additionally, developing financial navigation services and screening tools to identify patients at risk of financial hardship can help connect them with appropriate resources.^29^ By addressing these factors collectively, the economic impact on cancer patients and their families can be mitigated, ultimately improving their quality of life and treatment outcomes.^8,29^

Despite several strengths, including a large, nationally representative patient cohort, the findings of the current study should be interpreted with consideration of a few limitations. IBM MarketScan database includes individuals with employer-sponsored health insurance, so the results may not be generalizable to patients who are uninsured or covered by government programs. Additionally, like all observational studies, the results may be subject to bias from unmeasured confounding factors. IBM MarketScan database does not provide detailed data on oncologic variables (such as histology, grade, and stage) or social patient characteristics (including race, socioeconomic status, and marital status). The degree of employment disruption observed in the current dataset may be an underestimate due to selection bias stemming from our inclusion criteria and the nature of the MarketScan database. Moreover, the current study explored the possibility of tracking subsequent employment changes, including returns to baseline status. However, the number of individuals who reverted to their original employment category was very small and attempts to model these transitions yielded unstable estimates due to limited sample size. Notably, the study focused on working-age patients with continuous, employer-sponsored insurance coverage before and after diagnosis, representing a population with a high potential for employment retention. Since MarketScan primarily collects data from large employers, the majority of individuals in this study were likely protected by the Americans with Disabilities Act and the Family and Medical Leave Act and probably had access to paid sick leave. Nonetheless, even as an underestimate, the data provide a substantial indication of the significant employment challenges faced by patients with GI cancer.

In conclusion, GI cancer diagnosis was associated with a significant financial burden and an increased risk of employment disruption. This financial strain and work disruption underscore the need for targeted interventions and support mechanisms to alleviate the economic and occupational impacts of GI cancer. Addressing these challenges is crucial for improving patient quality of life and ensuring they receive the necessary support to manage both their health and financial well-being effectively.

## Supplementary Information

Below is the link to the electronic supplementary material.

Supplementary file1 (DOCX 16 KB)

## Data Sharing Statement

The data for this study was obtained from the IBM MarketScan database. There are restrictions to the availability of this data, which is used under license for this study. Data can be accessed with permission from the IBM MarketScan Commercial Database.
