# Supplementary material for: Employment Disruption and Financial Burden Associated with Gastrointestinal Cancers
Source: Ann Surg Oncol. 2025 Jun 26;32(10):7731–9. doi: 10.1245/s10434-025-17683-1 (PMC12454600; doi:10.1245/s10434-025-17683-1)
Supplement: Supplementary file 1 — Supplementary file1 (DOCX 16 KB) [file 10434_2025_17683_MOESM1_ESM.docx]

**Supplementary Table 1: International Classification of Diseases codes for identifying gastrointestinal cancers.**

| **Cancer type** | **ICD-9** | **ICD-10-CM** |
| --- | --- | --- |
| Esophagus | 150.0, 150.1, 150.2, 150.3, 150.4, 150.5, 150.8, 150.9 | C15.0, C15.1, C15.2, C15.3, C15.4, C15.5, C15.8, C15.9 |
| Stomach | 151.0, 151.1, 151.2, 151.3, 151.4, 151.5, 151.6, 151.8, 151.9 | C16.0, C16.1, C16.2, C16.3, C16.4, C16.5, C16.6, C16.8, C16.9 |
| Liver | 155.0, 155.1, 155.2, 155.9 | C22.0, C22.1, C22.2, C22.3,  C22.4, C22.7, C22.8, C22.9 |
| Pancreas | 157.0, 157.1,157.2, 157.3,157.4, 158.8, 157.9 | C25.0, C25.1, C25.2, C25.3,  C25.4, C25.7, C25.8, C25.9 |
| Gallbladder and extrahepatic biliary tract | 156.1, 156.8,156.9 | C23.0, C24.0, C24.8, C24.9 |
| Colorectal | 153.3, 153.4, 153.5, 153.6, 153.7, 153.8, 153.9, 154.0, 154.1, 154.2, 154.3, 154.8 | C18.0, C18.1, C18.2, C18.3, C18.4, C18.5, C18.6, C18.7, C18.8, C18.9, C19, C20 |
